# Polymeric Hydrogel Scaffolds: Skin Tissue Engineering and Regeneration

**DOI:** 10.34172/apb.2022.069

**Published:** 2021-09-14

**Authors:** Varuna Naga Venkata Arjun Uppuluri, Shanmugarajan Thukani Sathanantham, Sai Krishna Bhimavarapu, Lokesh Elumalai

**Affiliations:** Department of Pharmaceutics, School of Pharmaceutical Sciences, Vels Institute of Science, Technology & Advanced Studies (VISTAS), Chennai, 600 117, Tamil Nadu, India.

**Keywords:** Hydrogel, Skin, Tissue engineering, Wound healing

## Abstract

Tissue engineering is a novel regenerative approach in the medicinal field that promises the regeneration of damaged tissues. Moreover, tissue engineering involves synthetic and natural biomaterials that facilitate tissue or organ growth outside the body. Not surprisingly, the demand for polymer-based therapeutical approaches in skin tissue defects has increased at an effective rate, despite the pressing clinical need. Among the 3D scaffolds for tissue engineering and regeneration approaches, hydrogel scaffolds have shown significant importance for their use as 3D cross-linked scaffolds in skin tissue regeneration due to their ideal moisture retention property and porosity biocompatibility, biodegradable, and biomimetic characteristics. In this review, we demonstrated the choice of ideal biomaterials to fabricate the novel hydrogel scaffolds for skin tissue engineering. After a short introduction to the bioactive and drug-loaded polymeric hydrogels, the discussion turns to fabrication and characterisation techniques of the polymeric hydrogel scaffolds. In conclusion, we discuss the excellent wound healing potential of stem cell-loaded hydrogels and Nano-based approaches to designing hydrogel scaffolds for skin tissue engineering.

## Introduction

Every year several people worldwide receive several invasive procedures for the treatment and management of skin tissue defects. Even though several approaches support skin wound healing, all those approaches failed to mimic the microenvironment of the extracellular matrix. Recently the tissue engineering offered the ability to regenerate the tissue defects that the body fails to restore.^1,2^

**Figure 1:**
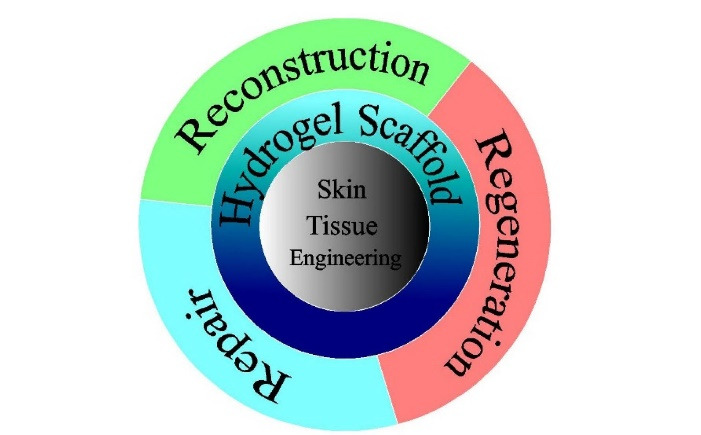
Role of hydrogel scaffolds in skin tissue engineering concepts.

In the medicinal field, tissue engineering is an innovative technique that guarantees the recovery of damaged tissue. In addition, tissue engineering includes the use of many biomaterials that facilitate the growth of the body’s tissues or organs. Moreover, synthetic and natural biomaterials play a significant role in the growth of tissues or organs by their extracellular matrix support and regenerative potential. Skin tissue, however, has taken the value of recent tissue engineering attempts and has also exhibited the proof of concept in clinical studies. Further, (as shown in Figure 1) the recent attempts in skin tissue engineering demonstrated the proof of concept in clinical studies for diabetic patients and burnt wound patients.^3-5^

Not surprisingly, the demand for polymer-based therapeutically approaches in skin tissue defects has increased at an effective rate, despite the pressing clinical need. While in the past, usage of inert polymers for tissue regeneration was quite common, however nowadays, polymer researchers moved to the fabrication of scaffolds by using polymeric materials, which integrate with biological molecules or cells and regenerate tissues. When applied to the skin, the polymeric hydrogel scaffolds must promote angiogenesis, reepithelialisation, collagen synthesis and integration into adjacent tissues without causing scarring.^6,7^

**Figure 2:**
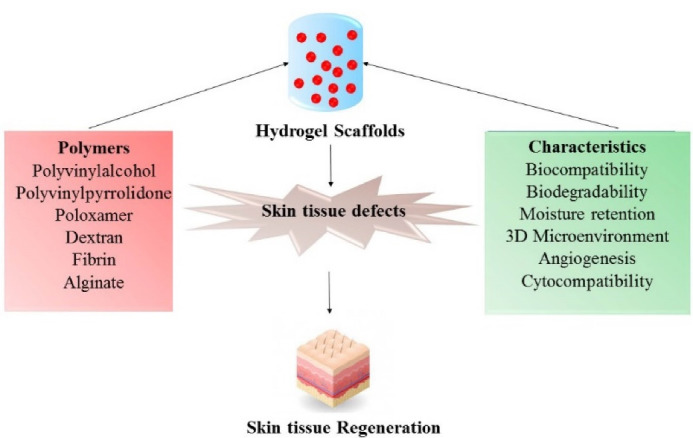
Depicts about the characteristics of various polymers that plays a significant role in skin tissue regeneration.

Although there have been many research works and reviews on hydrogel scaffolds for tissue regeneration, this review focuses mainly on the polymers involved in the fabrication of novel hydrogel scaffolds, specifically for use in skin tissue engineering (Figure 2). Furthermore, this paper points out stem cell-loaded hydrogels and nanoscale hydrogel scaffolds involved in skin wound healing concepts.

## Hydrogel scaffolds for skin tissue regeneration

**Figure 3:**
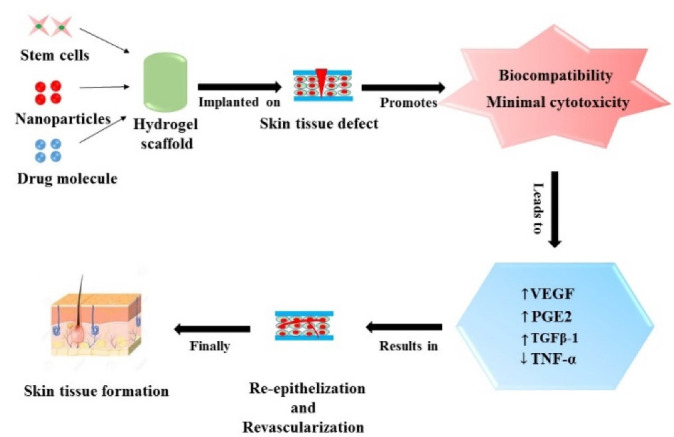
Represents role of stem cells, nanoparticles and drug molecules loaded polymeric hydrogels in skin tissue regeneration.

Hydrogels refer to the 3D cross-linked polymeric networks that can absorb and retain water molecules. However, the cross-linked networks prevented the hydrophilic polymeric chains from converting into an aqueous phase in the hydrogel scaffolds. The porosity of the hydrogel scaffold plays a significant role in the diffusion of the macromolecules such as nutrients and oxygen molecules during the lack of functional blood vessels.^8,9^ Further, several studies demonstrated that hydrogels with greater pore size were effective in cellular proliferation (i.e., Fibroblasts and Keratinocytes) and extracellular matrix regeneration. Finally, it appears that the incorporation of cells into the polymeric hydrogel scaffolds often enhances the ideal cellular distribution within the skin defect site^10^ (see Figure 3 and Table 1). Although, bioactive and drug-based polymeric hydrogel scaffolds offer many advantages, including minimal cytotoxicity, excellent swelling potential, biomimetic, bioadhesive, and biocompatibility characteristics. There are some additional challenges with delivering active pharmaceuticals, including limited hydrophobic drug distribution and too slow responses of stimuli-responsive hydrogels. Recent literature suggested the fabrication of nanoparticle-loaded hydrogels and faster-acting hydrogels (i.e., by making hydrogels thinner and smaller) to solve these issues. Whereas, in the case of bioactive polymeric hydrogels (such as dextran, fibrin, and alginate-based hydrogel scaffolds), inadequate mechanical strength limits the activity of these hydrogels. It is possible to address this limitation by newer fabrication strategies using both synthetic/ natural polymer-based hydrogels that act as an adequate replacement for wound care and wound management.^11,12^

**Table 1:** Role of polymeric hydrogels in skin tissue regeneration.

**Formulation**	**Author**	**Role**	**Reference**
PVA/Dextran-aldehyde composite hydrogel.	Zheng et al (2019)	• Fluid absorption (6 times of original weight), and tensile strength (5.6 MPa). • Interconnected porous networks (5–10 μm).	^13^
Gentamycin loaded PVA/sericin hydrogel.	Tao et al (2019)	• Excellent hydrophilicity, and swelling behavior.	^14^
Novel liposomal polyvinyl pyrrolidone hydrogel	Vogt et al (2001)	• Excellent tolerability and delivery characteristics	^15^
Novel lignin- CS- PVA composite hydrogel.	Zhang et al (2019)	• Ideal mechanical strength (tensile stress up to 46.87 MPa), and the protein adsorption capacity. • Reepithelization and Revascularization.	^16^
Icariin loaded PVA/agar hydrogel scaffold.	Uppuluri et al (2019)	• Biocompatibility and biomimetic characteristics.	^17^
Sodium fusidate loaded PVA/PVP film-forming hydrogel.	Kim et al (2015)	• Flexibility, elasticity, and also shown optimal drug release along with fast film forming ability.	^18^
PAA/CS and PVP.	Rasool et al (2019)	• Thermal stability, biodegrability and antibacterial activity (against *E. coli*).	^19^
Neomycin sulfate-loaded PVA/PVP/SA hydrogel.	Choi et al (2016)	• Bioadhesive strength, and tensile strength characteristics.	^20^
Poloxamer/CS/hyaluronic hydrogel loaded with antioxidant molecules (i.e. vitamins A, D, and E).	Soriano-Ruiz et al (2020)	• Ideal mechanical properties and antimicrobial potential.	^21^
Hyaluronic acid-poloxamer hydrogel.	Li et al (2019)	• Moisture retaining characteristics. • Anti-microbial activity.	^22^
WJ-MSC loaded SAP/PF127 hydrogel.	Deng et al (2020)	• Enhanced the collagen content, hair follicles.	^23^
hmCS and oxidized dextran hydrogel.	Du et al (2019)	• Viscoelasticity, non-cytotoxic and bioadhesive characteristics. • Antibacterial activity.	^24^
SA and GMs incorporated Dex-HA hydrogel.	Zhu et al (2018)	• Porosity (80%), swelling ratio (8 times in water and 7 times in PBS), antimicrobial potency. • Increased proliferation of NIH-3T3 fibroblast cells.	^25^
Dextran hydrogel.	Shen et al (2015)	• Anti- inflammatory response. • Angiogenesis and reepithelization.	^26^
Granule-lyophilised platelet-rich fibrin loaded PVA hydrogel scaffolds.	Xu et al (2018)	• Biodegradability (17–22%). • Mechanical strength (6.451×10^−2^MPa). • Re-epithelization and revascularization.	^27^
PEG-fibrin hydrogel	Burmeister et al (2017)	• Enhanced the granular tissue formation without delaying the reepithelization process.	^28^
Benlysta loaded sodium alginate hydrogel.	Wang et al (2020)	• Swelling rate (150%). • Sustained rate of drug release (i.e. 50% of release in 72 hours). • Biodegradability (i.e. retaining 95% weight within 72 hours).	^29^
PVA/modified sodium alginate hydrogel.	Wu et al (2020)	• Biomimetic property. • Minimal self-healing time (15 seconds).	^30^
Naringenin loaded alginate hydrogel.	Salehi et al (2020)	• Porosity (86.7 ± 5.3%). • Swelling (342 ± 18% at 240 min). • Sustained release profile (74.09 ± 8.71% over 14 days). • Biodegradability (89% at 14^th^ day). • Antibacterial property.	^31^

Abbreviations: PVA, polyvinyl alcohol; PVP, poly (N-vinyl-2-pyrrolidone); PAA, Poly acrylic acid; CS, chitosan; SA, sanguinarine; GMs, gelatin microsphere; hmCS, hydrophobically modified chitosan; HA, hyaluronic acid; WJ-MSC, Wharton's jelly mesenchymal stem cell; SAP, sodium ascorbyl phosphate.

Zheng et al fabricated the polyvinyl alcohol (PVA) and dextran-aldehyde (DA) hydrogel using freeze-thaw cycles and lyophilisation techniques. Due to their ideal fluid absorption (6 times of original weight), tensile strength (5.6 MPa), water vapour transmission rate (WVTR) (2100 g m^−2^ day^−1^), and interconnected porous networks (5–10 μm), the PVA/DA hydrogels promoted wound healing significantly, along with minimal hemolytic potential and cytotoxicity. Additionally, in a full-thickness skin defect model, PVA/DA hydrogel showed expertise in accelerating the wound healing cycle, enhancing wound contraction efficiency, and skin regeneration. All of these findings showed that these hydrogel dressings with several favourable functions for the wound healing process significantly promoted the healing of full-thickness skin wounds, suggesting possible application as wound dressings to cure skin tissue defects in the distant future.^13^

A non-toxic, super-absorbent, and antibacterial hydrogel as a skin wound dressing is of considerable importance.^14^ In order to develop a SS/PVA hydrogel, Tao et al blended the silk-sericin (SS) and PVA through repeated freeze-thaw cycles. Further, these developed SS/ PVA hydrogels photoluminescence indicated the ideal interaction of PVA with sericin. Given its porous structure, SS/PVA hydrogel showed ideal hydrophilicity and swelling behaviour. PVA blending improved sericin’s thermostability and mechanical property significantly yet did not affect sericin and PVA’s crystallinity. Besides, cytotoxicity and release studies of the Gentamicin loaded SS/PVA hydrogel likewise exhibited the ideal cytocompatibility on the mammalian cells and moderately released the Gentamycin from the SS/PVA hydrogel to hinder the bacterial development and retain the cell viability. Finally, this hydrogel showed incredible injury mending properties and end up being a phenomenal medication conveyance vehicle for forestalling topical wounds.

Despite improving reepithelialisation, moist wound treatment has a higher risk of bacterial infection. Therefore, Vogt et al developed a novel liposomal polyvinyl pyrrolidone hydrogel for enhancing the wound healing potential of patients with burnt skin defects. Moreover, the polyvinyl pyrrolidone-iodine liposomes act as an enhanced wound-moisture delivery system, release polyvinylpyrrolidone (PVP)-iodine at minimal rates, and engage with the wound surface to produce more effective cellular interactions. Furthermore, compared to wounds treated with a conventional antiseptic chlorhexidine-gauze, the wound treated with polyvinyl pyrrolidone-iodine liposome hydrogel showed ideal reepithelialisation, antibacterial and wound healing potential. Finally, the liposomal PVP-Iodine hydrogel’s excellent tolerability and delivery characteristics led to qualitative progress in treating burnt skin tissue defects.^15^

## Polymers involved in the development of hydrogel scaffolds for skin tissue regeneration

Because of their wide range of properties and bioactivity, synthetic and natural polymers have been popular biomaterials for creating these hydrogel scaffolds. Natural and synthetic polymers are two distinguished polymers that play a significant role in the skin tissue regeneration process (Table 2).Each natural polymer has its unique properties and uses depending on its‌‌‌n. In addition to their inherent biodegradability, natural polymer-based hydrogels are rich in biologically recognised moieties that stimulate cellular functions. In contrast to natural polymers, synthetic polymer-based hydrogels have enormous control over the structural properties, such as biodegradation, mechanical strength, and chemical response.^32,33^

**Table 2:** Advantages and Disadvantages of the polymers involved in fabrication of hydrogel scaffolds for skin tissue regeneration

**Name of the Polymer**	**Advantages**	**Disadvantages**	**Reference**
Polyvinyl alcohol	• Shown ideal biocompatibility, biodegradability, controlled rate of release. • Minimal cytotoxicity	• Poor cell adhesive characteristics. • Weak hydrogel durability at high temperature.	^34^
Polyvinylpyrrolidone	• Enhanced the permeation potential and biocompatibility of the incorporated therapeutical moieties.	• Poor mechanical characteristics. • Minimal swelling capacity.	^35^
Poloxamer	• Excellent biocompatibility, high solubilisation characteristics (in case of hydrophobic drugs).	• Poor biodegradability and mechanical characteristics.	^36^
Dextran	• Ideal bioadhesive property. • Promoted sustained rate of protein or drug release.	• High cost and faster degradability.	^37^
Fibrin	• Minimal risk of immunogenic response. • Controlled rate of degradation.	• Low mechanical stiffness.	^38^
Alginate	• Controlled release of therapeutical molecules. • Biomimetic property.	• Faster rate of degradation. • Minimal cellular adhesion.	^39^

## Synthetic polymers

### PVA

PVA is a synthetic hydrophilic polymer; its ideal biocompatibility, bioadhesive, and film-forming characteristics played a significant role in developing the hydrogel scaffolds for skin tissue engineering concepts.^16^ Zhang et al fabricated novel composite lignin – chitosan (CS) – PVA hydrogel has an ideal wound dressing, with excellent antioxidant and antibacterial activity. However, despite the excellent biocompatibility and antibacterial activity of PVA-CS composite hydrogel, its poor mechanical strength limits its application to wound dressings. Also, PVA-CS composite hydrogel cannot satisfy the injury dressing prerequisites as an environmental conditioner for speeding up the skin tissue regeneration process. Further, the sulfonate groups in the lignin’s structure shaped ionic bonds with amino groups in the CS, increases the hydrogel’s mechanical strength (tractable pressure up to 46.87 MPa), the protein adsorption limit, and the hydrogel’s ability to manage the wound environment. In a murine injury model, the lignin-CS-PVA hybrid hydrogel significantly worked on injury mending. Besides, the novel hydrogel offers new freedoms for exceptionally effective treatment of skin tissue defects.

Recently, tissue regeneration concepts had been a promising approach for the restoration of defective skin tissues. However, due to its 3D scaffolding nature and moisture-retaining property, topical hydrogels played a significant role in the burn wound healing process compared to other hydrogels involved in the skin tissue regeneration process.^17^ Uppuluri et al developed the icariin-loaded PVA/Agar hydrogel scaffold and implanted it on the burnt tissue defects for restoring the damaged extracellular matrix. The characterisation studies such as FTIR (Fourier transform infrared), differential scanning calorimetry, and X-ray powder diffraction revealed the chemical structure, melting enthalpy, and compatibility of the developed hydrogel scaffolds. Further, in the case of FESEM (field emission scanning electron microscopy*)* results, it was clearly shown that icariin exhibited ideal spreading in the interconnected network structure of the developed PVA/Agar hydrogel scaffolds. Additionally, the wound healing potential, confirmed at the end of 21 days by histopathological analysis, provided visual proof for the reepithelisation potential of icariin-based PVA/agar hydrogel scaffolds.

### PVP

PVP is a synthetic hydrophilic polymer with excellent biocompatibility, cross linkability, and absorption potential in response to various physical and chemical stimuli. Hence due to that reason, PVP was utilised in the formulation of hydrogel scaffolds. In order to promote a facile application and ideal wound healing, Kim et al developed a novel sodium fusidate-loaded film-forming hydrogel by using the PVA, PVP, Propylene glycol, and ethanol as additives. The 2/12 ratio of PVA/PVP in this study demonstrated the ideal flexibility, elasticity, optimal drug release, and fast film-forming ability at the administration site (i.e., 4 minutes). In addition, histopathological and stability studies showed that sodium fusidate-loaded film-forming hydrogel demonstrated excellent re-epithelisation, scarless tissue formation, and stability at 45°C for at least six months compared to the sodium fusidate-loaded commercial product. Hence, due to this reason, sodium fusidate-loaded film-forming hydrogel was identified as an ideal product with the facile application for wound treatment and management.^18^

Rasool et al, in the presence of 74% neutralised polyacrylic acid (PAA), successfully fabricated the CS and PVP based stimuli-responsive hydrogels for wound healing applications. Moreover, the FTIR spectra revealed the different functional groups and chemical interactions within the developed hydrogels. In contrast, the thermal analysis, biodegradation, and antimicrobial studies revealed thermal stability, biodegradability, and antibacterial activity (against *E.coli*) of the developed hydrogels. Further, at PH 8, when compared to other hydrogel samples, 0.5 g PVP loaded hydrogels showed ideal swelling (i.e., 10220%) in distilled water due to the diffusion of aqueous solution into the hydrogel matrix. Furthermore, the controlled rate of release (i.e., 91.2 %) of Ag-Sulfadiazine into the PBS within 80 minutes revealed the intra and inter-molecular interactions within 0.5 g PVP loaded hydrogels. Finally, in his study, the developed hydrogels were ideal drug delivery systems for wound-related applications.^19^

According to Choi et al, at a weight ratio of 1/10/0.8/0.8, the neomycin sulfate-loaded hydrogel composed of the drug molecule, PVA, PVP, and SA boosted the swelling capacity (502.3 ± 18.1%), bioadhesive strength (117.2 ± 4.5 g), and maximum tensile strength (38.2 ± 2.5 x 10 -3 N/mm^2^). Moreover, the weak interaction between the polymers and drug molecules resulted in 100% drug release within 4 hours in all formulations. Further, the hydrogel has provided a more enhanced wound healing effect than the commercial product, ensuring that granulation tissue disappears and returning the wound tissue to normal. Hence, due to this reason, the neomycin sulfate-loaded hydrogel was found to be a promising pharmaceutical product for the treatment and management of skin tissue defects.^20^

### Poloxamer

Due to its ideal non-toxic, localised drug delivery, biodegradable, biocompatible, and thermoreversible characteristics, the Poloxamer 407 acted as a highly effective polymer to formulate a hydrogel scaffold meant for skin tissue regeneration. Despite considerable progress in wound dressing development, wound management poses a significant challenge, forcing the patient and health care community to bear a significant burden. Mainly due to its complex pathophysiology, wounds with bacterial growth present significant challenges to traditional wound dressings, and therefore nowadays, the development of novel and more effective wound healing methods was rapidly increased. For example, Soriano-Ruiz et al fabricated the poloxamer (POL)/CS/hyaluronic hydrogel loaded with antioxidant molecules (i.e., vitamins A, D, and E) to ameliorate skin burns therapy. Moreover, pH studies revealed that the acidic pH of the hydrogel (i.e., 4.6±0.1) resulted in bacterial inhibition and minimised the time taken for wound healing. Further, the physicochemical and biological properties-based studies revealed that the developed hydrogel system demonstrated the ideal mechanical properties for its importance in skin wounds with antimicrobial and wound healing potential, being harmless to the normal skin. Finally, this vitamin-loaded hydrogel was a therapeutically promising component to investigate its wound healing potential in future clinical trials.^21^

Recently skin tissue defects and wound healing research was focused primarily on post-trauma haemostasis, infection prevention, cutaneous regeneration, and angiogenesis. Nonetheless, air permeability and moisture retention, other factors crucial to wound healing, received relatively little attention.^22^ Li et al designed a hyaluronic acid‐poloxamer (HA‐POL) hydrogel and evaluated its wound healing efficacy in the case of the rat model. Further, the research findings demonstrated that excellent air permeability and moisture retention properties of these biodegradable HA-POL hydrogels, in turn, prevented microbial infection and minimised the time taken for wound healing, and thus avoided the usage of antibiotics. Furthermore, histopathological findings revealed that compared to bFGF treated rats, HA‐POL hydrogel enhanced the revascularisation, angiogenesis, and reepithelisation process enhanced the skin tissue repair process. Finally, HA‐POL hydrogel proved to be an optimal material for treating and managing skin tissue defects.

Factors such as inadequate grafting, insufficient retention time minimised the therapeutic efficacy for wound regeneration. Therefore these issues need to be investigated in order to overcome them. For example, Deng et al in their study demonstrated the role of Wharton’s jelly mesenchymal stem cell (WJ-MSC) loaded sodium ascorbyl phosphate (SAP) - Pluronic F-127 (PF-127) hydrogel in case of full-thickness skin wound model. In addition, the 8th-day *in-vivo* study results demonstrated that WJ-MSC loaded SAP- PF-127 hydrogel enhanced collagen content and hair follicles and facilitated skin tissue regeneration with minimal scar formation. Finally, the immune histochemical analysis revealed the anti-inflammatory and angiogenic potential of WJ-MSC-loaded SAP- PF-127 hydrogel.^23^

## Natural polymers

### Dextran

Due to its effective biocompatibility, biodegradability, and minimal toxicity characteristics, dextran played a significant role in tissue regeneration studies. However, the optimal release characteristics of the dextran resulted in the utilisation of this polymer in hydrogel scaffold formulation. Here Du et al demonstrated the hydrophobic interaction of hydrophobic aliphatic chains in the case of the recently fabricated modified chitosan (hmCS) and oxidised dextran hydrogel. The characterisation studies like FESEM, rheology, cytotoxicity, and tissue adhesive tests revealed the ideal morphology, viscoelasticity, non-cytotoxic, and bioadhesive characteristics of these developed hydrogels. Further, the antibacterial studies on the *S. aureus* and *P. aeruginosa* revealed that at the total bacterial concentration (108 CFU/mL), the developed hydrogels have shown killing efficacy of 95.0% and 96.4%. Finally, upon observing the wound healing model, it was proved that the developed hydrogels had shown excellent regenerative potential in skin tissue defects. In addition, this paved the way for the use of this hydrogel in other tissue regeneration approaches.^24^

Numerous risks associated with burnt wounds (such as infection and pathogenic scar tissue formation) play a crucial role in delaying the wound closure and increasing wound-related complications.^25^ Zhu et al developed the sanguinarine (SA) gelatin microsphere (GMs) incorporated dextran-hyaluronic acid (Dex-HA) hydrogel that showed the ideal porosity (80%), swelling ratio (8 times in water and seven times in PBS), increased proliferation of NIH-3T3 fibroblast cells and maintained SA release profile in the GM. Moreover, the *in vitro* degradation, and antimicrobial testing revealed the faster degradation (31% in PBS and 24% in hyaluronidase), and antimicrobial potency of the SA GMs incorporated Dex-HA hydrogel. Further, the histopathological, Masson’s trichome, and immune histochemical findings revealed the reepithelisation, revascularisation of the hydrogel-treated groups. Finally, SA GMs incorporated hydrogel proved to be effective in burnt skin tissue regeneration with minimal scar formation.

Recently, skin tissue grafts and skin transplants meant for burn skin wound healing were fraught with risks such as improper revascularisation and reepithelisation. Moreover, synthetic therapy facilitated wound healing in a regenerative manner includes a non-immunogenic, off-the-shelf strategy to enhance the clinical care of burnt skin tissue defects.^26^ Shen et al demonstrated the effective burnt wound healing mechanisms of the dextran-based hydrogel. The purpose of the model was to analyse the clinical translation of hydrogel therapy and its mechanisms of tissue regeneration response. Further, the dextran-based hydrogel’s initial anti-inflammatory response stimulated the angiogenesis followed by excellent reepithelisation to promote the effective skin tissue regeneration process. Finally, dextran-based hydrogel promoted effective skin tissue regeneration paved the way for clinical trials to enhance the treatment of patients with severe burns.

### Fibrin

In the past, topical wounds are typically treated and managed with traditional dressings.^27^ Xu et al fabricated a novel granule-lyophilised platelet-rich fibrin-loaded polyvinyl alcohol hydrogel scaffolds as an ideal wound dressing material for skin tissue regeneration. Notably, the characterisation studies revealed the excellent morphology, biocompatibility, biodegradability (17–22%), and mechanical strength (6.451×10^−2^ MPa) of this granule-lyophilised platelet-rich fibrin loaded polyvinyl alcohol hydrogel scaffolds. Finally, the *in vivo*, histopathological and immune histochemical analysis revealed the ideal granular tissue formation, reepithelisation, and revascularisation in the granule-lyophilised platelet-rich fibrin loaded hydrogel scaffolds, indicating its possible applications as an ideal dressing for topical wounds.

Recently, rather than autografts and allografts, the tissue-engineered hydrogels gained profound importance in burnt wound healing concepts because of their ideal biocompatibility and biomimetic characteristics.^28^ Burmeister et al developed the fibrin-based hydrogel for accelerating the tissue regeneration activity in burnt wounds. Further, the histopathological and histochemical studies revealed that fibrin hydrogels enhanced the granular tissue formation without delaying the reepithelisation process. Finally, compared to other skin substitutes, the immunomodulatory effects of PEG- Fibrin hydrogels played a significant role in fastening the burnt wound healing process.

During the severe burns, the lack of accessibility of typical skin tissue sources resulted in a fatality in the patients.^40^ In order to overcome this problem, Natesan et al developed a debrided skin adipose stem cells (dsASCs) loaded collagen-polyethylene glycol (PEG) fibrin-based bilayer hydrogel for the treatment of severe burns. The novelty of this concept lies within the isolation of stem cells from the patient’s burnt wound site, which further enhances the tissue compatibility of these hydrogels. Finally, the *in vitro* and *in vivo* studies facilitate the evidence for the utilisation of the dsASCs loaded hydrogels in the treatment of skin tissue defects.

### Alginate

Due to its ideal deflocculating, gelling, protein absorption potential, biocompatibility, moisture retention, biodegradable and viscoelastic characteristics, the anionic polysaccharide like alginate played a significant role in a hydrogel formulation. Furthermore, the wound healing process is one of the most complex biological mechanisms that result in fibrotic tissue mass formation without hormone activity for the skin tissue defects caused by trauma and burns. Therefore, Wang et al developed a benlysta loaded sodium alginate hydrogel with excellent anti-inflammatory and skin tissue regeneration characteristics. Further, gelation experiments involving benlysta loaded sodium alginate hydrogels revealed that the sodium alginate ratio could control the gelation time, suggested its potential use as both skin dressing and subcutaneous injection. Furthermore, the characterisation studies revealed the ideal swelling rate (150%), sustained rate of drug release (i.e., 50% of release in 72 hours), biodegradability (i.e., retaining 95% weight within 72 hours), excellent fibroblast and epidermal cellular proliferation of the benlysta loaded sodium alginate hydrogel. Eventually, this benlysta loaded sodium alginate hydrogel will give new ideas for the treatment and management of skin tissue defects.^29^

Recently, self-healing hydrogels as a replenishable substance had received considerable interest. Besides, the poor biocompatibility and long self-healing duration of the traditional self-healing hydrogel limited their application in skin tissue regeneration.^30^ Therefore, Wu et alfabricated a polyvinyl alcohol/ modified sodium alginate hydrogel with excellent self-healing ability and minimal self-healing time (i.e., 15 seconds). However, maintaining the flexibility, good electrical conductivity, and cold resistance property NaCl and glycerol were utilised to fabricate the self-healing hydrogels. Finally, the ideal biomimetic property of self-healing polyvinyl alcohol/modified sodium alginate hydrogel resulted in the triggering of capacitor screens of the electronic products.

Many researchers often consider wounds as a significant physical liability. Hence due to this reason, several types of research were performed on the topical wounds to find an ideal method for the acceleration of the topical wound healing process. However, due to their adequate and appropriate characteristics, polymeric hydrogels played a significant role in the skin tissue regeneration process.^31^ Salehi et al fabricated a naringenin-loaded alginate hydrogel for accelerating the skin tissue regeneration process in case of skin tissue defects. Further, the *in vitro* and *in vivo* characterisation studies revealed that ideal porosity (86.7 ± 5.3%), swelling (342 ± 18% at 240 minutes), Sustained release profile (74.09 ± 8.71% over 14 days), biodegradability (89% at 14th day), and antibacterial property of the 20% naringenin-loaded alginate hydrogel significantly enhance the reepithelisation, revascularisation, and collagen synthesis at the site of skin tissue defects. Finally, the findings of this experiment provided significant proof that 20% naringenin-loaded alginate hydrogel had a practical impact on skin tissue defects.

## Fabrication of the polymeric hydrogel scaffolds

Appropriate cross-linking techniques will enable a hydrogel scaffold to possess the desired architecture and mechanical characteristics. This section will cover the most common cross-linking techniques to fabricate hydrogel scaffolds‌‌‌‌.^41^

## Physical cross-linking

The hydrogels typically result from reversible intramolecular interactions, such as ionic/electrostatic interactions, hydrogen bonds, and hydrophobic/hydrophilic‌‌interactions, referred to as physically cross-linked hydrogels. One of the most crucial advantages of physical cross-linking techniques is their biological safety. Since no chemical cross-linking agents are necessary, it ultimately results in hydrogel scaffolds with minimal cytotoxicity. Additionally, a physically cross-linked hydrogel is stimuli-responsive, self-healing, and injectable at‌‌‌temperature. Finally, using this cross-linking technique, it is possible to design bioactive hydrogels that are enough to encapsulate living cells and deliver‌‌cules.^42^

## Chemical cross-linking

Compared with physically cross-linked hydrogels, chemically cross-linked hydrogels usually have covalent bonds among polymer chains, and most of their linkages are generally more substantial and more permanent than their counterparts. So far, these chemically cross-linked hydrogels have resulted from polymerisation-induced cross-linking, enzyme induced cross-linking, Diels-Alder “click” reactions, Schiff bases, etc. Further, chemically cross-linked hydrogels, on the other hand, are generally more stable under physiological conditions and exhibit exceptional mechanical characteristics and tunable degradation behaviour.^43^

## Characterisation of the polymeric hydrogel scaffolds

### Porosity

**Figure 4:**
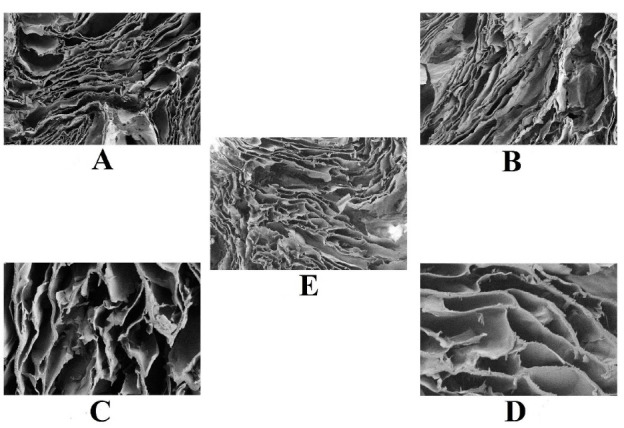
Represents the porosity within the PVA/agar polymeric hydrogel scaffolds. (A) represents FESEM image focused at 100 µm and magnification was about 250X. (B) represents image at 100 µm with magnification 250X. (C) represents image at 20 µm with magnification 1000X. (D) represents the image at 20 µm with magnification 1000X. E. represents the image at 100 µm with magnification 264X.^44^

A highly porous and interconnected network-like structure (i.e. visualised by FESEM technique) is necessary for a hydrogel scaffold to facilitate angiogenesis and cellular proliferation in skin tissue defects. While fabricating a hydrogel scaffold, researchers must consider crucial parameters like the average pore size, pore volume, interconnectivity, and its distribution. This porous, biocompatible network of the hydrogel scaffold promotes the new tissue formation and serves as a temporary template for their‌‌‌rganisation (Figure 4). Additionally, it is also essential for the pores to have the right size since too small pores would not allow cells to penetrate and create a new skin extracellular matrix, which would hinder angiogenesis.^44^

### Biocompatibility

In tissue engineering, biocompatibility refers to a scaffold’s ability to accommodate cellular activity and the transmission of molecular and mechanical signals. In addition to their chemistry, structure, and morphology, scaffolds’ biocompatibility is affected by the methods used to synthesise them, process them, and sterilise them. Recent research has shown that several biodegradable polymers, including PVA, PVP, Poloxamer, Dextran, Fibrin, and Alginate, are suitable for various medical applications due to their excellent‌.^45^

## Stem cells in skin tissue regeneration

A stem cell is a distinct undifferentiated cell that can self-renew and differentiate into a particular cell lineage. Recently, (as shown in Figure 3 and Table 3) stem cells derived from various tissues with varying differentiation and tissue regenerative potential were utilised in hydrogel scaffolds to treat skin tissue defects. They have shown considerable potential for enhancing wound healing rate and consistency in the skin. The endogenous factors obtained from the MSCs enhance angiogenesis by modifying the inflammatory response and promoting wound repair. However, incorporating the MSCs spheroids into biomaterial upregulated the tropical factor secretion and enhanced regeneration by focusing the cells at the burnt skin tissue defect site.^46^ Murphy et al developed a fibrin hydrogel delivery system to enhance the therapeutical efficacy of the MSC spheroids by improving the neovascularisation and anti-inflammatory property of the incorporated MSCs spheroids. However, the manipulation of the four input variables of the multifactorial statistical analysis designed for fibrin hydrogel revealed that stiffness of the hydrogel plays a significant role in MSC spheroids delivery, i.e., a slight increase in stiffness of the fibrin hydrogel enhanced the secretion of the endothelial factors by decreasing the TNF-α secretion. Thereby, it results in cellular proliferation, macrophage polarisation, angiogenesis, and reepithelisation in the hydrogel-treated groups. Finally, the statistical analysis involved in fabricating the fibrin hydrogel played a significant role in enhancing skin tissue regeneration.

**Table 3:** Significance of Stem cells loaded hydrogels in skin tissue regeneration

**Formulation**	**Author**	**Significance**	**Reference**
dsASCs loaded collagen- PEG fibrin-based bilayer hydrogel.	Natesan et al (2013)	• Isolation of stem cells from the burnt wound site of the patient enhanced the tissue compatibility of these hydrogels.	^40^
Mesenchymal stem cell spheroids loaded Fibrin hydrogel.	Murphy et al (2017)	• Excellent viscoelastic properties. • Optimal release of mesenchymal stem cells. Enhanced the VEGF (promotes the neovascularization) and PGE2 (modulates both inflammatory and fibrogenesis phase) secretion. • Decreased TNF-α secretion induces fibroblasts to produce proteoglycan and fibronectin in the injured tissues, promoting extracellular matrix formation.	^46^
ASCs loaded Pluronic F127 hydrogel.	Kaisang et al (2017)	• Ideal biomimetic. • 3D scaffold characteristics. • Increased TGF-β1 secretion contributes to wound healing by inhibiting inflammation, promoting angiogenesis, and collagen synthesis and deposition.	^47^
Silver sulfadiazine and adipose stem cells loaded fibrin hydrogel.	Banerjee et al (2019)	• Controlled release of silver sulfadiazine and adipose stem cells. • Minimal cytotoxicity.	^48^
Adipose stem cells loaded polyethylene glycol-fibrin hydrogels.	Burmeister et al (2018)	• Ideal contraction. • Angiogenesis.	^49^

Abbreviations: dsASCs, debrided skin adipose stem cells; PEG, polyethylene glycol; ASCs, Adipose-derived stem cells.

In diabetes, skin tissue defect healing poses a significant challenge because of impaired angiogenesis. Even though there are several formulations for diabetic wound healing, the lack of reepithelisation and revascularisation resulted in poor diabetic healing.^47^ Kaisang et al developed the novel adipose-derived stem cells (ASCs) loaded pluronic F127 hydrogel to alleviate diabetic wounds. Due to its ideal biomimetic and 3D scaffold characteristics, pluronic F127 hydrogel successfully incorporated and released the stem cells at the site of tissue defects. Additionally, the immunohistochemical staining (CD31) and immunofluorescence (Ki-67) staining and gene expression (growth factor) studies revealed that stem cells loaded Pluronic F127 hydrogel enhanced the diabetic wound healing by promoting the ideal reepithelisation, angiogenesis, and keratinocyte proliferation in the damaged portion. Finally, ASC-loaded hydrogel proved to be an ideal therapeutical approach for diabetic wound healing.

Usually, the ideal burnt wound healing requires effective infection control.^48^ In order to achieve this, Banerjee et al developed an antimicrobial fibrin hydrogel loaded with silver sulfadiazine and adipose stem cells for effective burn wound healing. Rapid degradation, minimal inflammation characteristics of the fibrin polymer (derived from fibrinogen) resulted in using this polymer to formulate the hydrogel scaffolds meant for skin tissue regeneration. Further, the two-treatment cycle of this fibrin hydrogel resulted in controlled release of silver sulfadiazine followed by delivery of the adipose stem cells, which helps to minimise silver toxicity associated with traditional topical delivery approaches. Further, the* in vivo* and immunohistochemistry findings revealed the ideal neovascularisation, reepithelisation in the SSD-CSM-ASC-FPEG (silver sulfadiazine/CS microspheres/ASC/ PEGylated fibrin gel) hydrogel treated groups. Finally, the SSD-CSM-ASC-FPEG hydrogel played a crucial role in decreasing bacterial infection along with ideal neovascularisation and burnt skin tissue regeneration.

In case of total body surface area burns, autograft harvesting results in morbidities on the donor site. In this condition, meshing grafts increased the potential of contraction and hypertrophic scarring, restrict the range of motion, and exacerbates cosmesis. However, many tissue engineering-based approaches had acclaimed the importance of the ASC in burnt skin tissue defects.^49^ Burmeister et al demonstrated the efficacy of the ASCs loaded polyethylene glycol-fibrin hydrogels as an adjunct to meshed autografts in the porcine skin wound model. Moreover, the histopathological and immune histochemical analyses revealed that when delivered with meshed autografts, the ASC-loaded glycol-fibrin hydrogels showed ideal contraction and angiogenesis in the case of the porcine skin wound model. Finally, meshed autografts delivered with ASC-loaded hydrogels are clinically crucial in providing effective and efficient treatment to reduce donor sites and promote skin tissue regeneration.

## Nano based approach for skin tissue regeneration

Complications with the traditional drug delivery systems resulted in the advancement of nanotechnology-driven therapeutic interventions, resulted in acceleration of the healing process, and ultimately led to a complete restoration of skin tissue defects (as shown in Figure 3 and Table 4). Khampieng et al effectively manufactured a 1 and 5 mM silver nanoparticle-inserted PVP hydrogel utilising the γ-illumination at 25, 35 45 kGy. The results of the transmission electron microscopy and scanning electron microscopy with an energy dispersive x-ray analysis uncovered the spherical shape (~4–10 nm) and the distribution of silver nanoparticles within the developed hydrogels. Further, the PVP hydrogels embedded with 5 mM nAg shown ideal cumulative release, antibacterial activity and limited the contact time for obtaining the 99% bacterial reduction. Besides, 5 mM Silver nanoparticles - implanted PVP hydrogels not just gave an ideal moist wound recuperating climate yet averted the bacterial infection and improved the wound healing process additionally.^50^

**Table 4:** Importance of the nanoparticles loaded hydrogels in skin tissue regeneration.

**Formulation**	**Author**	**Importance**	**Reference**
Silver nanoparticle-embedded PVP hydrogel	Khampieng et al (2014)	• Anti-bacterial activity at the site of damaged skin tissue.	^50^
Sodium alginate/ polyvinyl alcohol hydrogel loaded with 5-hydroxymethyl furfural and silver nanoparticles.	Kong et al (2019)	• Antioxidant potential. • Moisture retaining property.	^51^
Nano silver incorporated PVP/ Carrageenan hydrogel.	Singh et al (2015)	• Hindered the fluid accumulation during exudation of wounds.	^52^

Treatment of large acute or chronic wounds remains challenging due to the lack of appropriate strategies to improve skin tissue regeneration. While growth factor-based wound products are successful, they are expensive and potentially associated with increased mortality from cancer. Hence due to this reason, there was a need for the design and development of reliable, safe, and ideal strategies to tackle large or chronic wounds.^51^ Kong et al fabricated the sodium alginate/polyvinyl alcohol hydrogel loaded with 5-hydroxymethylfurfural and silver nanoparticles for accelerating the wound healing process in the case of skin tissue defects. Further, the physicochemical characterisation studies revealed the ideal biocompatibility, non-cytotoxic, controlled rate of drug release, anti-inflammatory and antibacterial properties of the 5-hydroxymethylfurfural and silver nanoparticles incorporated sodium alginate/polyvinyl alcohol hydrogel. Additionally, the *in vivo* studies, histopathological and immune histochemical staining confirmed the excellent reepithelisation, revascularisation, and angiogenic potential of 5-hydroxymethylfurfural and silver nanoparticles loaded hydrogel. Eventually, due to its excellent antioxidant and anti-inflammatory potential 5-hydroxymethylfurfural and silver nanoparticles loaded hydrogel played a significant role in accelerating the wound healing process of skin tissue defects.

Singh et al developed the nanosilver incorporated PVP/carrageenan hydrogel using the gamma irradiation technique. Moreover, the outcome of his study revealed that the introduction of 100 ppm nanosilver into the hydrogel effectively hindered microbial growth. Additionally, the fluid absorption capacity and moisture vapour transmission rate studies of the nano silver-based hydrogels demonstrated the hydrogels’ ability to hinder fluid accumulation during skin tissue defects. Finally, the ideal characteristics of the nanocomposite loaded hydrogel paved the way for the utilisation of these hydrogel scaffolds in skin tissue engineering and biomedical applications.^52^

## Conclusion and perspective

Hydrogels are optimistic scaffolds for skin tissue engineering, owing to their minimal invasiveness and biomimetic characteristics. Biomaterials such as PVA, PVP, poloxamer, dextran, fibrin and alginate are the most often used polymers to fabricate hydrogel scaffolds, owing to their ideal mechanical properties. This review highlighted hydrogel scaffolds fabricated by a significant variety of biomaterials for skin tissue engineering applications.

Since the past few years, several studies have focused on developing novel hydrogel scaffolds for skin tissue repair. However, to achieve effective skin tissue regeneration, there are still several significant challenges to overcome in designing the hydrogel scaffold. The major obstacle to fabricating hydrogel scaffolds for skin tissue engineering is the design of the bioactive polymeric scaffolds with ideal mechanical properties, minimal cytotoxicity for cellular proliferation, nutrient transportation and drug delivery. Additionally, the novel multifunctional polymeric hydrogel scaffold should be prepared with ideal therapeutic molecules that act on multi-target signalling mechanisms and accelerate the regenerative potential in skin tissue defects. Therefore, the development of multifunctional hydrogels plays a significant role in addressing the current challenges and prospects in clinical aspects of regenerative medicine.

## Acknowledgments

The authors are thankful to Vels Institute of Science, Technology & Advanced Studies (VISTAS), Chennai, for the facilities extended.

## Ethical Issues

This article does not contain any animal experimentation performed by any of the authors.

## Conflict of interest

The authors declare that they have no conflict of interest.
